# Effectiveness of BNT162b2 and CoronaVac vaccines in preventing SARS-CoV-2 Omicron infections, hospitalizations, and severe complications in the pediatric population in Hong Kong: a case-control study

**DOI:** 10.1080/22221751.2023.2185455

**Published:** 2023-03-15

**Authors:** Vincent Ka Chun Yan, Franco Wing Tak Cheng, Celine Sze Ling Chui, Francisco Tsz Tsun Lai, Carlos King Ho Wong, Xue Li, Eric Yuk Fai Wan, Joshua Sung Chih Wong, Esther Wai Yin Chan, Ian Chi Kei Wong, Mike Yat Wah Kwan, Patrick Ip

**Affiliations:** a Centre for Safe Medication Practice and Research, Department of Pharmacology and Pharmacy, Li Ka Shing Faculty of Medicine, The University of Hong Kong, Hong Kong, People’s Republic of China; b School of Nursing, Li Ka Shing Faculty of Medicine, The University of Hong Kong, Hong Kong, People’s Republic of China; c School of Public Health, Li Ka Shing Faculty of Medicine, The University of Hong Kong, Hong Kong, People’s Republic of China; d Laboratory of Data Discovery for Health (D24H), , Hong Kong Science and Technology Park, Sha Tin, Hong Kong, People’s Republic of China; e Department of Family Medicine and Primary Care, School of Clinical Medicine, Li Ka Shing Faculty of Medicine, The University of Hong Kong, Hong Kong, People’s Republic of China; f Department of Medicine, School of Clinical Medicine, Li Ka Shing Faculty of Medicine, The University of Hong Kong, Hong Kong, People’s Republic of China; g Department of Paediatrics and Adolescent Medicine, Princess Margaret Hospital, Hong Kong, People’s Republic of China; h Aston Pharmacy School, Aston University, Birmingham, UK; i Department of Paediatrics and Adolescent Medicine, Hong Kong Children's Hospital, Hong Kong, People's Republic of China; j Department of Paediatrics and Adolescent Medicine, The University of Hong Kong, Hong Kong, People’s Republic of China

**Keywords:** COVID-19, vaccine effectiveness, pediatric population, mRNA vaccine, inactivated vaccine

## Abstract

Severe COVID-19 appears to be disproportionately more common in children and adolescents since the emergence of Omicron. More evidence regarding vaccine effectiveness (VE) is urgently needed to assist policymakers in making decisions and minimize vaccine hesitancy among the public. This was a case-control study in the pediatric population using data extracted from the electronic health records database in Hong Kong. Individuals aged 3–17 with COVID-19 confirmed by polymerase chain reaction were included in the study. Each case was matched with up to 10 controls based on age, gender, and index date (within 3 calendar days). The VE of BNT162b2 and CoronaVac in preventing COVID-19, hospitalizations, and severe outcomes were estimated using conditional logistic regression adjusted by patients’ comorbidities and medication history during the outbreak from January to August 2022. A total of 36,434 COVID-19 cases, 2231 COVID-19-related hospitalizations, and 1918 severe COVID-19 cases were matched to 109,004, 21,788, and 18,823 controls, respectively. Compared to the unvaccinated group, three doses of BNT162b2 or CoronaVac was associated with reduced risk of infection [VE: BNT162b2: 56.0% (95% CI: 49.6–61.6), CoronaVac: 39.4% (95% CI: 25.6–50.6)], hospitalization [VE: BNT162b2: 58.9% (95% CI: 36.1–73.6), CoronaVac: 51.7% (11.6–73.6)], and severe outcomes [VE: BNT162b2: 60.2% (95% CI: 33.7–76.1), CoronaVac: 42.2% (95% CI: −6.2–68.6)]. Our findings showed that three doses of BNT162b2 or CoronaVac was effective in preventing COVID-19, hospitalizations, and severe outcomes among the pediatric population during Omicron-dominant pandemic, which was further enhanced after a booster dose.

## Key points

In this territory-wide case–control study, three doses of BNT162b2 or CoronaVac were associated with reduced risk of infection [Vaccine Effectiveness (VE): BNT162b2: 56.0%, CoronaVac: 39.4%], hospitalization (VE: BNT162b2: 58.9%; CoronaVac: 51.7%), and severe outcomes (VE: BNT162b2: 60.2%; CoronaVac: 42.2%).

## Background

In the pre-Delta pandemic, children and adolescents were less likely to experience severe COVID-19 [1]. On the contrary, the emergence of SARS-CoV-2 Omicron variant appears to disproportionately cause more severe symptoms in children and adolescents, including more hospital admissions, convulsion, croup, and multisystem inflammatory syndrome in children (MIS-C) [2,3]. According to the data from the COVID-19-Associated Hospitalization Surveillance Network (COVID-NET), the weekly COVID-19-associated hospitalization rates in younger patients aged 17 or below increased by 4-fold from 1.8 per 100,000 population during the Delta predominance period to 7.1 per 100,000 during Omicron predominance period compared to a 2.5-fold increase in adults from 15.5 to 38.4 per 100,000 [2,4]. Although the side effect profiles of COVID-19 vaccines are now becoming established based on extensive pharmacovigilance studies [5,6], more evidence regarding vaccine effectiveness (VE) is urgently needed, especially during the Omicron era, which will assist policymakers in making decisions and to minimize vaccine hesitancy among the public.

Hong Kong Special Administrative Region (HKSAR), China, started a territory-wide vaccination programme in February 2021 using mRNA vaccine BNT162b2 (Comirnaty, BioNTech/Pfizer/Fosun) and inactivated vaccine CoronaVac (Sinovac Biotech HK Limited). The vaccination programme was extended to adolescents aged 12–15 starting in June 2021 and was further extended to children aged 5–11 in January 2022. The minimum age for the CoronaVac vaccination was further lowered to infants aged 3 years in February 2022 and aged 6 months in August 2022.

The VE of mRNA vaccines has been demonstrated in children and adolescents in several trials conducted in the early phase of the Omicron pandemic when BA.1 and BA.2 were the dominant subvariants [7–10]. CoronaVac is one of only a few approved vaccines for the pediatric population and is widely used in developing countries. It has been demonstrated to have a VE of around 40% against symptomatic COVID-19 and 60% against hospitalization in children based on two observational studies in Brazil and Chile during the Omicron period. However, these studies did not compare the VE of mRNA and inactivated vaccines, and did not consider the effects of booster doses [11,12]. Therefore, we conducted a territory-wide case–control study to examine the effectiveness of BNT162b2 and CoronaVac vaccines in children and adolescents in Hong Kong during the current Omicron-dominated pandemic, which also considered the number of vaccine doses and their effectiveness on a range of clinical outcomes.

## Methods

### Data sources

Clinical data on COVID-19 vaccinations in the pediatric and adolescent population in Hong Kong were obtained from the electronic health records database of the Hospital Authority (HA), the vaccination records of the Department of Health (DH), and COVID-19-confirmed case records from the Centre of Health Protection (CHP). Anonymized unique patient identifiers were used to integrate these databases. The HA is a statutory administrative organization that manages all public inpatient services and most of the public outpatient services in Hong Kong. The electronic health records database contains data on patient demographics, diagnoses, prescriptions, and laboratory tests, which provides real-time information to support routine clinical management across all clinics and hospitals in the HA. The DH maintains a vaccination records database of all individuals in Hong Kong. The CHP maintains a database of all confirmed COVID-19 cases based on both mandatory and voluntary reporting of positive polymerase chain reaction (PCR) and rapid antigen test (RAT) results in Hong Kong. These population-based databases have been used in studies on the risk of adverse effects of COVID-19 vaccinations and in other COVID-19 pharmacovigilance studies [5,6,13–27].

### Study design and population

This is a case-control study conducted on children and adolescents aged 3–17 years in Hong Kong. The study period was from 1 January to 15 August, 2022. Individuals with an incident COVID-19-related outcome during the study period were identified as cases. Controls were selected from all other individuals without a COVID-19-related outcome who attended HA services during the study period. The index date was the date of COVID-19-related outcomes for cases and the attendance date for controls. Subjects in the control group who reported a positive RAT result on the online voluntary reporting platform were excluded. The matching procedures were conducted separately for each of the COVID-19-related outcomes. Each case was matched with up to 10 controls according to age, sex, index date (within 3 calendar days), and Charlson Comorbidity Index (0, 1-2, 3-4, ≥5) [28].

### Definitions of vaccine exposure

Two COVID-19 vaccines, BNT162b2 and CoronaVac, are provided by the Hong Kong government free of charge in its mass vaccination programme. The BNT162b2 vaccine was first made available to individuals aged ≥16 in March 2021, and was extended to adolescents aged 12–15 in June 2021 and to children aged 5–11 in February 2022. The CoronaVac vaccine was first made available to adolescents aged 12–17 in November 2021 and was extended to children aged 5–11 in January 2022. Details of the full rollout schedule is listed in Supplementary Table 1. Individuals have a choice of the first vaccine dose between BNT162b2 or CoronaVac, but are then restricted to the same vaccine for the second dose. For the booster vaccination, individuals have a choice of either vaccine. In this study, COVID-19 vaccination status was classified into eight mutually exclusive groups based on the type and number of vaccine doses administered: (i) 1 dose BNT162b2, (ii) 1 dose CoronaVac, (iii) 2 doses BNT162b2, (iv) 2 doses CoronaVac, (v) 3 doses (all BNT162b2), (vi) 3 doses (all CoronaVac), (vii) 3 doses (2 doses BNT162b2 and CoronaVac booster), and (viii) 3 doses (2 doses CoronaVac and BNT162b2 booster). Individuals who received a fourth dose or with incomplete vaccination records were excluded from the study.

### Definitions of COVID-19 and outcomes

The outcomes investigated in this study were (i) COVID-19 diagnosis; (ii) COVID-19-related hospitalization within 28 days after COVID-19 diagnosis; and (iii) severe COVID-19 defined as any diagnosis of complications or requiring procedures (including ventilatory support) listed in Supplementary Table 2, prescription of tocilizumab, methylprednisolone or intravenous immunoglobulin G, admission to an intensive care unit (ICU), or death within 28 days after COVID-19 diagnosis.

COVID-19 diagnosis was defined as a positive PCR result obtained from the CHP of the HKSAR government and/or HA databases. A positive PCR result is recognized as the gold-standard test for COVID-19 given its high specificity of >99% [29]. The Hong Kong government has implemented extensive PCR testing for SARS-CoV-2 in public hospitals and clinics for those presenting with COVID-like symptoms and for close contacts of confirmed cases. The government has also set up territory-wide community testing centres to screen asymptomatic individuals and provide regular testing for staff at high risk of exposure to SARS-CoV-2, such as those working in nursing homes. A sensitivity analysis was conducted where voluntarily reported positive RAT cases were also included in the definition for COVID-19. Information regarding all-cause mortality was extracted from the Hong Kong Deaths Registry, the official governmental registry covering all registered deaths in Hong Kong.

### Statistical analysis

Conditional logistic regressions adjusted for pre-existing asthma, diabetes, epilepsy, and use of immunosuppressants within 90 days were applied to calculate the crude and adjusted odds ratio (OR) with 95% confidence interval [30]. Vaccine effectiveness (VE) was estimated by (1 – adjusted OR) × 100%. Subgroup analyses were conducted and were stratified by age (3–11 and 12–17 years). To evaluate waning VE, additional analyses were conducted at five time-since-vaccination intervals (0–13, 14–60, 61–120, 121–180, and ≥180 days) after receiving each vaccine dose. For each time-since-vaccination interval, only eligible matched pairs, in which both cases and controls were either unvaccinated or fell within the specific time-since vaccination interval, were included to derive the corresponding estimates. Two sensitivity analyses were conducted. First, cases and controls who received their last vaccine dose for more than 90 days were excluded. Second, both PCR and RAT positive cases were recognized in the definition of COVID-19.

All statistical tests were two-sided and *P* values less than 0.05 were considered statistically significant. Statistical analyses were conducted using R version 4.0.3 (www.R-project.org). Two investigators (VY and FC) conducted the statistical analyses independently for quality assurance. STROBE (Strengthening the Reporting of Observational Studies in Epidemiology) statement checklists were followed to guide the transparent reporting in this case–control study.

### Ethical approval

This study was approved by the Central Institutional Review Board of the Hospital Authority of Hong Kong (CIRB-2021-005-4) and the Department of Health Ethics Committee (LM171/2021).

## Results

**Figure 1.**
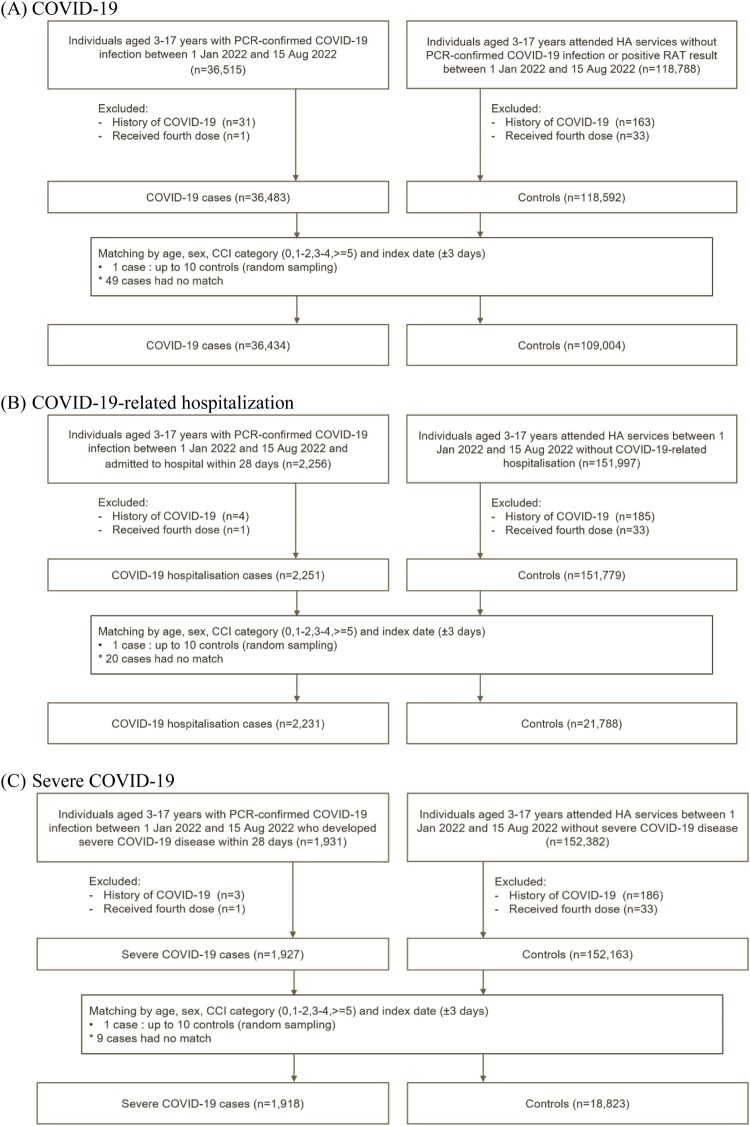
Selection of cases and controls.

From 1 January to 15 August 2022, a total of 36,434 COVID-19 cases, 2231 COVID-19-related hospitalizations, and 1918 severe COVID-19 cases were matched to 109,004, 21,788 and 18,823 controls, respectively (Figure 1). The baseline characteristics of cases and controls are summarized in Table 1. During the study period, eight COVID-19-related deaths and 99 cases of COVID-19-related ICU admission or ventilatory support were identified, which are summarized in Supplementary Table 3. Among the eight COVID-19-related deaths (mean [SD] age of 8.75 [5.06] years and 50% male), six (75%) were unvaccinated, one (12.5%) was vaccinated with one dose of BNT162b2, and one (12.5%) was vaccinated with one dose of CoronaVac.

**Table 1.** Baseline characteristics of cases and controls.

Outcomes	Infection		Hospitalization		Severe COVID-19	
	Cases	Controls	Cases	Controls	Cases	Controls
Number of individuals	36,434	109,004	2231	21,788	1918	18,823
Age, years (mean (SD))	10.35 (4.00)	10.35 (4.00)	8.70 (4.38)	8.67 (4.38)	8.53 (4.31)	8.50 (4.30)
Sex, male (%)	20766 (57.0)	62211 (57.1)	1283 (57.5)	12561 (57.7)	1106 (57.7)	10871 (57.8)
Charlson Comorbidity Index (mean (SD))	0.03 (0.19)	0.03 (0.18)	0.07 (0.34)	0.03 (0.20)	0.05 (0.30)	0.03 (0.19)
Time since recent dose (mean (SD))	88.72 (80.59)	75.75 (80.79)	85.07 (67.14)	81.05 (72.60)	84.41 (66.22)	79.38 (69.90)
Comorbidities – no. (%)						
Asthma	789 (2.2)	1955 (1.8)	53 (2.4)	467 (2.1)	40 (2.1)	333 (1.8)
Diabetes	45 (0.1)	251 (0.2)	9 (0.4)	46 (0.2)	7 (0.4)	32 (0.2)
Epilepsy	237 (0.7)	1961 (1.8)	83 (3.7)	200 (0.9)	77 (4.0)	180 (1.0)
Medication use within 90 days – no. (%)						
Immunosuppressants	36 (0.1)	223 (0.2)	13 (0.6)	43 (0.2)	9 (0.5)	37 (0.2)

**Figure 2.**
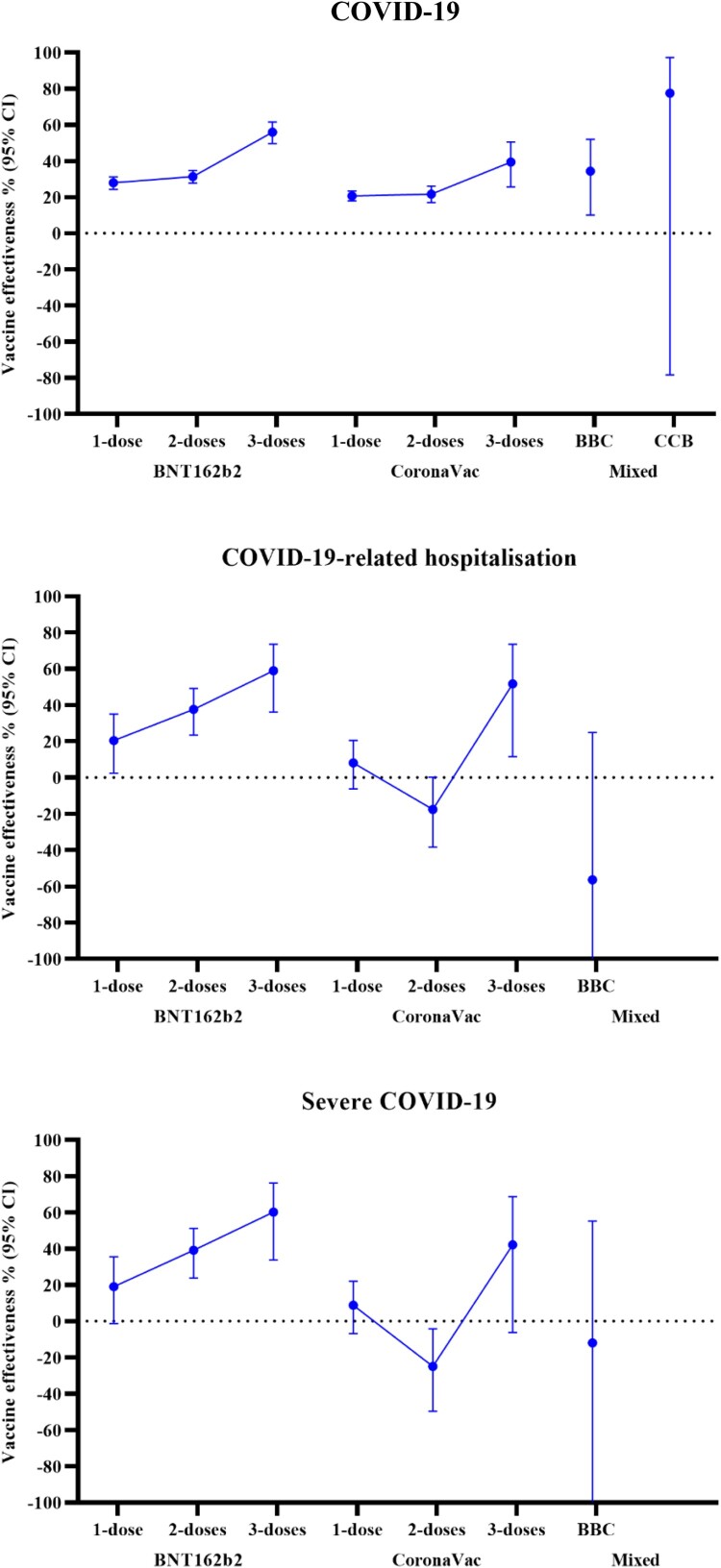
Vaccine effectiveness among children and adolescents with different vaccination status.

A dose–response relationship was observed in the VE against COVID-19, in which VE increased with more vaccine doses (Table 2 and Figure 2). In children and adolescents who received two vaccine doses, VE (95% CI) was 31.3% (27.8; 34.7) for BNT162b2 and 21.7% (17.0; 26.2) for CoronaVac. In children and adolescents who received three vaccine doses, VE was observed to be higher at 56.0% (49.6; 61.6) for BNT162b2 and 39.4% (25.6; 50.6) for CoronaVac.

**Table 2.** Vaccine effectiveness among children and adolescents with different vaccination status.

Vaccination status	Case	Control	Crude OR (95% CI)	Adjusted OR (95% CI)	VE% (95% CI)
*COVID-19*					
Unvaccinated	17,825	48,738	(Ref)	(Ref)	(Ref)
*1 dose*					
BNT162b2	4100	13,241	0.733 (0.700–0.769)	0.721 (0.688–0.756)	27.9 (24.4; 31.2)
CoronaVac	6011	20,016	0.800 (0.772–0.828)	0.793 (0.766–0.821)	20.7 (17.9; 23.4)
*2 doses*					
All BNT162b2	5401	17,121	0.704 (0.670–0.741)	0.687 (0.653–0.722)	31.3 (27.8; 34.7)
All CoronaVac	2522	7595	0.793 (0.748–0.842)	0.783 (0.738–0.830)	21.7 (17.0; 26.2)
*3 doses*					
All BNT162b2	389	1660	0.449 (0.392–0.514)	0.440 (0.384–0.504)	56.0 (49.6; 61.6)
All CoronaVac	130	463	0.619 (0.505–0.759)	0.606 (0.494–0.744)	39.4 (25.6; 50.6)
B-B-C	55	161	0.674 (0.492–0.922)	0.656 (0.480–0.899)	34.4 (10.1; 52.0)
C-C-B	1	9	0.229 (0.029–1.814)	0.225 (0.028–1.783)	77.5 (−78.3; 97.2)
*COVID-19-related hospitalization*					
Unvaccinated	1188	11,181	(Ref)	(Ref)	(Ref)
*1 dose*					
BNT162b2	157	1631	0.758 (0.619–0.928)	0.796 (0.650–0.975)	20.4 (2.5; 35.0)
CoronaVac	290	2993	0.902 (0.781–1.042)	0.919 (0.795–1.062)	8.1 (−6.2; 20.5)
*2 doses*					
All BNT162b2	202	2494	0.588 (0.480–0.720)	0.624 (0.509–0.766)	37.6 (23.4; 49.1)
All CoronaVac	343	2753	1.147 (0.975–1.348)	1.175 (0.998–1.383)	−17.5 (−38.3; 0.2)
*3 doses*					
All BNT162b2	29	478	0.385 (0.248–0.599)	0.411 (0.264–0.639)	58.9 (36.1; 73.6)
All CoronaVac	12	213	0.477 (0.261–0.872)	0.483 (0.264–0.884)	51.7 (11.6; 73.6)
B-B-C	10	43	1.511 (0.727–3.140)	1.563 (0.751–3.250)	−56.3 (−225.0; 24.9)
C-C-B	0	2	–	–	–
*Severe COVID-19*					
Unvaccinated	1044	9956	(Ref)	(Ref)	(Ref)
*1 dose*					
BNT162b2	128	1307	0.780 (0.623–0.976)	0.809 (0.646–1.013)	19.1 (−1.3; 35.4)
CoronaVac	247	2596	0.897 (0.767–1.049)	0.912 (0.779–1.068)	8.8 (−6.8; 22.1)
*2 doses*					
All BNT162b2	165	2079	0.586 (0.469–0.732)	0.609 (0.488–0.762)	39.1 (23.8; 51.2)
All CoronaVac	294	2306	1.231 (1.029–1.474)	1.249 (1.042–1.497)	−24.9 (−49.7; −4.2)
*3 doses*					
All BNT162b2	22	360	0.379 (0.228–0.631)	0.398 (0.239–0.663)	60.2 (33.7; 76.1)
All CoronaVac	12	182	0.584 (0.318–1.072)	0.578 (0.314–1.062)	42.2 (−6.2; 68.6)
B-B-C	6	35	1.107 (0.444–2.764)	1.120 (0.448–2.799)	−12.0 (−179.9; 55.2)
C-C-B	0	2	–	–	–

OR: odds ratio, VE: vaccine effectiveness, CI: confidence interval, B-B-C: two doses of BNT162b2 followed by CoronaVac, C-C-B: two doses of CoronaVac followed by BNT162b2.

A similar dose–response relationship was observed for the VE of BNT162b2 against COVID-19-related hospitalizations and severe COVID-19. In children and adolescents who received BNT162b2, VE (95% CI) was 37.6% (23.4; 49.1) against COVID-19-related hospitalization and 39.1% (23.8; 51.2) against severe COVID-19 after two doses, which increased to 58.9% (36.1; 73.6) and 60.2% (33.7; 76.1) after three doses. For CoronaVac, there was a significant reduction in the risk for COVID-19-related hospitalizations and a trend toward a reduction for severe COVID-19 after three doses, with VE (95% CI) of 51.7% (11.6; 73.6) and 42.2% (−6.2; 68.6), respectively. However, there was no observed risk reduction after two doses (adjusted OR [95% CI] for hospitalization: 1.175 [0.998–1.383]; severe COVID-19: 1.249 [1.042–1.497]).

**Figure 3.**
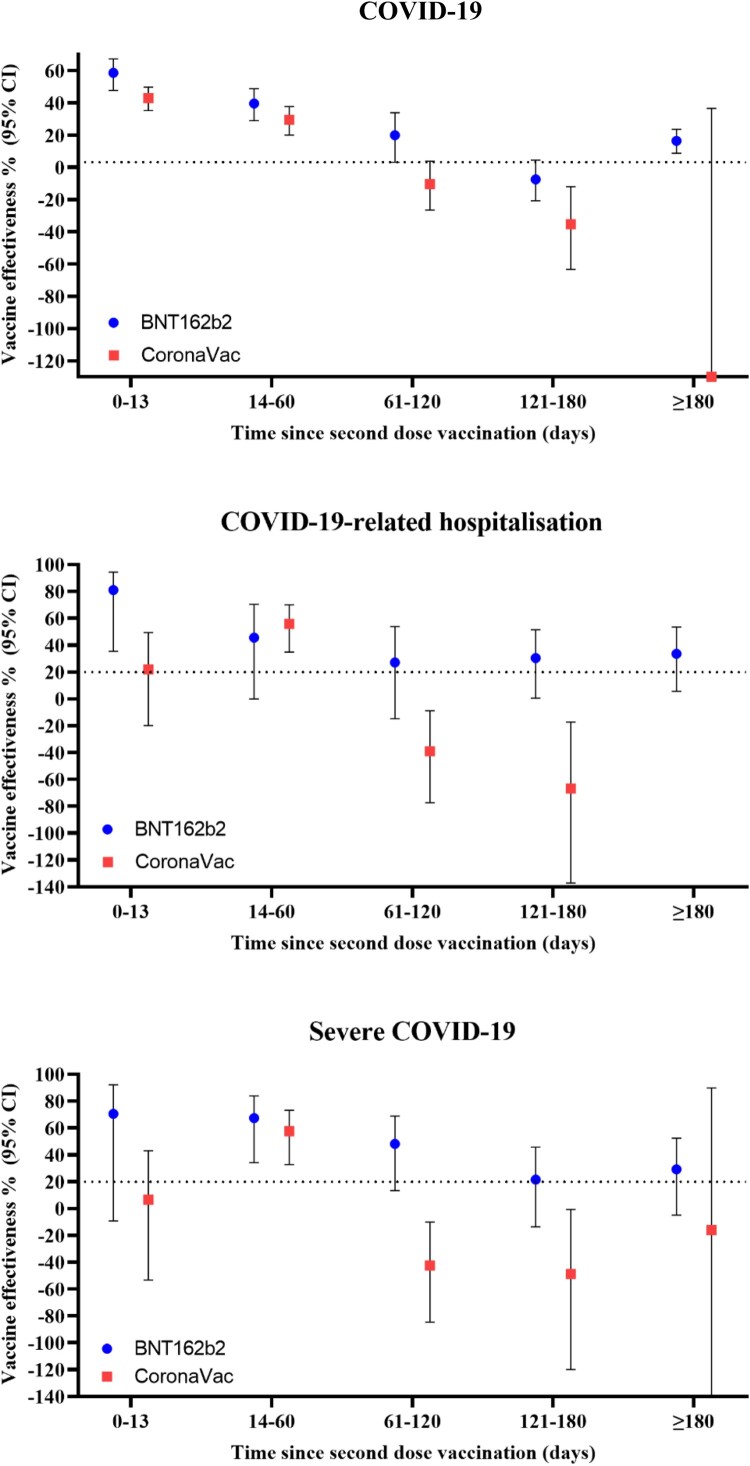
Vaccine effectiveness for different time-since-vaccination intervals after second dose.

A significant waning of effectiveness against all outcomes was observed for both vaccines over time (Table 3 and Figure 3). For BNT162b2, VE against COVID-19 and severe COVID-19 peaked at 0–13 days after the second dose, which remained effective up to 61–120 days. For CoronaVac, VE against COVID-19, hospitalization, and severe COVID-19 peaked at 14–60 days, with no significant risk reduction by 61–120 days. However, the trend of waning effectiveness after the third dose was less clear due to the limited number of children and adolescents receiving a third dose for more than 60 days. When waning effectiveness was accounted for by restricting the analyses to those who received their last vaccine dose within 90 days, there was a significant risk reduction in COVID-19-related hospitalizations after two and three doses of CoronaVac (VE [95% CI] two doses: 21.5 [2.1; 37.1], three doses: 50.9% [2.7; 75.2]), and a significant risk reduction against severe COVID-19 with three doses of CoronaVac (VE [95% CI]: 49.4% [1.5; 74.0]) (Supplementary Table 4).

**Table 3.** Vaccine effectiveness for different time-since-vaccination intervals.

Days since vaccine dose	VE % (95% CI)				
	0–13	14–60	61–120	121–180	≥180
*COVID-19*					
Unvaccinated	(Ref)	(Ref)	(Ref)	(Ref)	(Ref)
*1 dose*					
BNT162b2	57.9 (53.7–61.8)	28.9 (21.3–35.8)	−6.0 (−26.1–10.8)	−34.1 (−50.4 to −19.5)	−5.6 (−21.7–8.4)
CoronaVac	35.1 (32.0–38.0)	−6.3 (−12.5 to −0.5)	29.3 (−1.7–50.8)	26.9 (−6.2–49.6)	–
*2 doses*					
All BNT162b2	58.7 (47.8–67.3)	39.7 (29.0–48.7)	20.0 (3.2–33.9)	−7.4 (−20.7–4.4)	16.5 (8.7–23.6)
All CoronaVac	43.0 (35.2–49.8)	29.5 (20.1–37.7)	−10.3 (−26.5–3.8)	−35.2 (−63.3 to −12.0)	–
*3 doses*					
All BNT162b2	78.3 (63.4–87.1)	39.1 (13.1–57.3)	24.8 (−2.3–44.7)	15.6 (−26.8–43.8)	–
All CoronaVac	38.6 (−26.9–70.3)	−1.4 (−57.6–34.8)	33.9 (−49.4–70.8)	76.7 (−97.5–97.3)	–
B-B-C	49.3 (−53.3–83.2)	54.8 (−21.7–83.2)	−13.6 (−118.7–41.0)	11.0 (−158.8–69.4)	–
C-C-B	–	–	–	–	–
*COVID-19-related hospitalization*					
Unvaccinated	(Ref)	(Ref)	(Ref)	(Ref)	(Ref)
*1 dose*					
BNT162b2	19.2 (−25.2–47.8)	45.7 (18.2–64.0)	0.0 (−64.1–39.1)	−0.4 (−42.7–29.4)	26.8 (−52.6–64.9)
CoronaVac	7.5 (−13.7–24.7)	−3.4 (−27.3–15.9)	55.1 (9.7–77.6)	70.6 (34.7 to −86.8)	–
*2 doses*					
All BNT162b2	81.0 (35.2–94.4)	45.5 (−0.2–70.3)	27.1 (−14.9–53.7)	30.4 (0.4–51.3)	33.6 (5.5–53.3)
All CoronaVac	22.0 (−20.0–49.3)	55.8 (34.9–70.0)	−39.0 (−77.4 to −8.9)	−66.9 (−137.3 to −17.3)	–
*3 doses*					
All BNT162b2	91.9 (36.4–99.0)	58.9 (−15.4–85.4)	46.7 (−11.0–74.4)	62.0 (−43.3–89.9)	–
All CoronaVac	22.4 (−190.8–79.3)	69.5 (12.8–89.3)	−91.0 (−745.5–56.8)	–	–
B-B-C	–	–	–	–	–
C-C-B	–	–	–	–	–
*Severe COVID-19*					
Unvaccinated	(Ref)	(Ref)	(Ref)	(Ref)	(Ref)
*1 dose*					
BNT162b2	10.9 (−40.6–43.6)	47.8 (15.0–68.0)	23.2 (−32.7–55.6)	−6.9 (−55.4–26.5)	20.2 (−80.3–64.7)
CoronaVac	3.0 (−20.2–21.7)	−6.5 (−35.6–16.3)	39.8 (−22.7–70.5)	65.7 (22.0–84.9)	–
*2 doses*					
All BNT162b2	70.5 (−9.4–92.1)	67.3 (34.1–83.8)	48.0 (13.4–68.8)	21.4 (−13.6–45.7)	29.2 (−4.9–52.2)
All CoronaVac	6.5 (−53.4–43.0)	57.6 (32.8–73.2)	−42.5 (−84.6 to −10.1)	−48.8 (−120.0 to −0.7)	–
*3 doses*					
All BNT162b2	–	73.3 (20.5–91.0)	75.9 (43.6–89.7)	76.2 (−37.9–95.9)	–
All CoronaVac	58.8 (−58.5–89.3)	75.7 (23.7–92.3)	−33.8 (−463.6–68.2)	–	–
B-B-C	–	–	–	–	–
C-C-B	–	–	–	–	–

OR: odds ratio, VE: vaccine effectiveness, CI: confidence interval, B-B-C: two doses of BNT162b2 followed by CoronaVac, C-C-B: two doses of CoronaVac followed by BNT162b2.

Consistent results were observed between those aged 3–11 and 12–17 years (Table 4), and when RAT-positive cases were included in the definition of COVID-19 (Supplementary Table 5).

**Table 4.** Subgroup analyses by age.

Vaccination status	Aged 3–11			Aged 12–17		
	Case	Control	VE % (95% CI)	Case	Control	VE% (95% CI)
*COVID-19*						
Unvaccinated	14,560	40,045	(Ref)	3265	8693	(Ref)
*1 dose*						
BNT162b2	579	3256	55.0 (50.6; 59.0)	3521	9985	11.9 (6.3; 17.1)
CoronaVac	4766	16,069	22.3 (19.1; 25.3)	1245	3947	17.7 (11.1; 23.8)
*2 doses*						
All BNT162b2	174	742	45.3 (34.1; 54.5)	5227	16,379	23.1 (18.4; 27.5)
All CoronaVac	1571	4587	17.5 (10.5; 24.0)	951	3008	23.5 (16.3; 30.1)
*3 doses*						
All BNT162b2	0	0	–	389	1660	52.0 (44.8; 58.2)
All CoronaVac	67	259	39.1 (19.3; 54.1)	63	204	35.6 (13.2; 52.2)
B-B-C	0	0	–	55	161	28.0 (1.3; 47.5)
C-C-B	1	2	−16.0 (−1184.2; 89.5)	0	7	–
*COVID-19-related hospitalization*						
Unvaccinated	1009	9817	(Ref)	179	1364	(Ref)
*1 dose*						
BNT162b2	36	460	23.3 (−10.1; 46.5)	121	1171	23.1 (0.2; 40.8)
CoronaVac	249	2612	6.3 (−9.7; 20.0)	41	381	19.4 (−16.9; 44.4)
*2 doses*						
All BNT162b2	15	273	44.7 (3.4; 68.4)	187	2221	40.3 (23.6; 53.3)
All CoronaVac	270	2225	−23.9 (−49.8; −2.5)	73	528	3.8 (−34.0; 31.0)
*3 doses*						
All BNT162b2	0	0	–	29	478	61.9 (39.3; 76.0)
All CoronaVac	7	141	51.9 (−5.3; 78.0)	5	72	54.0 (−19.5; 82.3)
B-B-C	0	0	–	10	43	−45.9 (−207.1; 30.7)
C-C-B	0	0	–	0	2	–
*Severe COVID-19*						
Unvaccinated	899	8826	(Ref)	145	1130	(Ref)
*1 dose*						
BNT162b2	32	378	17.1 (−22.0; 43.7)	96	929	20.3 (−6.8; 40.6)
CoronaVac	217	2279	6.0 (−11.4; 20.7)	30	317	27.8 (−11.2; 53.2)
*2 doses*						
All BNT162b2	14	276	48.4 (8.1; 71.0)	151	1803	39.5 (20.6; 53.9)
All CoronaVac	235	1936	−24.4 (−53.1; −1.1)	59	370	−20.0 (−75.7; 18.1)
*3 doses*						
All BNT162b2	0	0	–	22	360	60.7 (32.6; 77.0)
All CoronaVac	7	127	47.6 (−15.3; 76.2)	5	55	35.5 (−70.8; 75.6)
B-B-C	0	0	–	6	35	−8.7 (−177.6; 57.5)
C-C-B	0	0	–	0	2	–

OR: odds ratio, VE: vaccine effectiveness, CI: confidence interval, B-B-C: two doses of BNT162b2 followed by CoronaVac, C-C-B: two doses of CoronaVac followed by BNT162b2.

## Discussion

Our study evaluated the real-world effectiveness of mRNA (BNT162b2) and inactivated virus (CoronaVac) COVID-19 vaccines among the pediatric population in Hong Kong during the Omicron-dominant pandemic. The results showed a clear dose–response relationship between the number of vaccine doses received and the level of vaccine effectiveness against COVID-19, hospitalizations, and severe symptoms. There was a reduction in VE starting from 60 days after the second dose of both vaccines, which highlights the need for booster doses in children and adolescents.

Our VE estimates during the Omicron predominant wave were generally lower than those in other studies, even after booster doses. For BNT162b2, we observed a VE of 31.3% against infection after two doses and a VE of 56.0% after a third dose compared to the reported VE ranging from 48% to 65% in two-dose BNT162b2 recipients aged 5–11 in the Singapore and Israel studies [7,10]. Our results for the VE against COVID-19-related hospitalizations were also lower than those reported by other studies. In the Singapore study, VE against hospitalization was reported to be higher than 80%. A similar study in the US that stratified individuals based on variants and age groups also reported higher VE estimates against hospitalization (Aged 12-17: 40% vs. 23.1%; Aged 5-11: 68% vs. 45.3%) [8]. For CoronaVac, our results showed VE against infection and hospitalization were 21.7% and −17.5% after two doses, and 39.4% and 51.7% after three doses, whereas VE estimates after two doses were 40% and 60% in the Brazil and Chile studies, respectively [11,12]. One potential explanation for the lower VE levels in our study is the waning protection of both BNT162b2 and CoronaVac, which has been observed in both pediatric and adult populations [9,31]. The VE against infection peaked during the first 60 days after vaccination and then declined thereafter regardless of the number of vaccines doses. Previous studies demonstrated sustained protection against severe outcomes despite waning protection against infection in the general population [31]. However, our results revealed the protection against hospitalization and severe outcomes started to diminish 2 months after vaccination, with more rapid waning in those receiving CoronaVac compared to BNT162b2. Another potential explanation was the introduction of the Vaccine Pass in Hong Kong, which limits unvaccinated individuals from visiting high-risk premises. As a result, the incidence of COVID-19 was underestimated in the unvaccinated group, and thus VE was also underestimated.

The mechanism of immune protection may account for a more rapid reduction in VE against COVID-19 over time, but to a lesser extent against COVID-19-related complications. Although humoral immunity mediated by antibodies blocks SARS-CoV-2 from entering host cells and thus prevents infection [32], specific CD4+ and CD8+ T cells appear to be responsible for limiting disease severity [33]. As a result, despite the rapid reduction of serum antibody titers, memory T cells are more durable and may contribute to the protection from severe disease.

The main strength of this study is that it is one of the first to provide real-world evidence on the effectiveness of both mRNA (BNT162b2) and inactivated virus (CoronaVac) vaccines against different Omicron subvariants among the pediatric population. Our findings highlight the importance of booster doses for reducing the risk of COVID-19 and preventing subsequent COVID-19-related complications including hospitalization and severe outcomes. Nonetheless, the findings of this study need to be interpreted with the following caveats. First, given the limited number of individuals receiving heterologous booster following the primary vaccine course, the VE of a booster dose against several outcomes could not be fully evaluated in this study. Second, a negative case-control study design is not feasible because only positive PCR or RAT results are reported to the DH. Moreover, there is a possibility that people with asymptomatic COVID-19 could be misclassified as controls, leading to a bias in the estimates towards the null. Asymptomatic COVID-19 cases cannot be captured unless nationwide screening is conducted, which at present is not possible in most countries. Furthermore, it is also possible that people with COVID-19 were misclassified due to false negative PCR results, but this is likely to be minimal due to the high specificity, and even less likely for severe cases. Thirdly, although the time-since-vaccination interval was not adjusted in our primary analysis, the assessment of waning vaccine effectiveness was stratified. Lastly, only patients who attended HA services could be included as control in our study. However, the selection bias was minimized during the matching process and with the adjustment of comorbidities during conditional logistic regression.

In conclusion, three doses of either BNT162b2 or CoronaVac vaccine is effective in preventing COVID-19, hospitalizations, and severe outcomes in children and adolescents during the Omicron variant-dominant pandemic. Our findings highlight the need for booster doses to enhance vaccine effectiveness in children and adolescents.

## Conflict of interest

FTTL has been supported by the RGC Postdoctoral Fellowship under the Hong Kong Research Grants Council and has received research grants from the Food and Health Bureau of the Government of the Hong Kong Special Administrative Region, outside the submitted work. XL has received research grants from the Food and Health Bureau of the Government of the Hong Kong Special Administrative Region; research and educational grants from Janssen and Pfizer; internal funding from the University of Hong Kong; and consultancy fees from Merck Sharp & Dohme, unrelated to this work. EYFW has received research grants from the Food and Health Bureau of the Government of the Hong Kong Special Administrative Region, and the Hong Kong Research Grants Council, outside the submitted work. CKHW reports receipt of research funding from the EuroQoL Group Research Foundation, the Hong Kong Research Grants Council, and the Hong Kong Health and Medical Research Fund; outside of the submitted work. EWYC reports honorarium from Hospital Authority; and grants from Research Grants Council (RGC, Hong Kong), Research Fund Secretariat of the Food and Health Bureau, National Natural Science Fund of China, Wellcome Trust, Bayer, Bristol-Myers Squibb, Pfizer, Janssen, Amgen, Takeda, and Narcotics Division of the Security Bureau of the Hong Kong Special Administrative Region, outside the submitted work. CSLC has received grants from the Food and Health Bureau of the Hong Kong Government, Hong Kong Research Grant Council, Hong Kong Innovation and Technology Commission, Pfizer, IQVIA, MSD, and Amgen; and personal fees from PrimeVigilance; outside the submitted work. ICKW reports research funding outside the submitted work from Amgen, Bristol-Myers Squibb, Pfizer, Janssen, Bayer, GSK, Novartis, the Hong Kong Research Grants Council, the Food and Health Bureau of the Government of the Hong Kong Special Administrative Region, National Institute for Health Research in England, European Commission, and the National Health and Medical Research Council in Australia; has received speaker fees from Janssen and Medice in the previous 3 years; and is an independent non-executive director of Jacobson Medical in Hong Kong. All other authors declare no competing interests. PI has received funding from the Hong Kong Research Grants Council, the Food and Health Bureau of the Government of the Hong Kong Special Administrative Region, and Hong Kong Jockey Club Charities Trust.

## Supplementary Material

Supplemental Material

## Data sharing statement

Data will not be available for others as the data custodians have not given permission.
