# Supplementary material for: Effectiveness of BNT162b2 and CoronaVac vaccines in preventing SARS-CoV-2 Omicron infections, hospitalizations, and severe complications in the pediatric population in Hong Kong: a case-control study
Source: Emerg Microbes Infect. 2023 Mar 15;12(1):2185455. doi: 10.1080/22221751.2023.2185455 (PMC10026771; doi:10.1080/22221751.2023.2185455)
Supplement: Supplemental Material [file TEMI_A_2185455_SM5671.docx]

**Supplementary Appendix**

**Supplementary Table 1.** Vaccination program priority groups rollout schedule in Hong Kong

| Order of expansion | Date of rollout | Vaccination group |
| --- | --- | --- |
| First^1^ | Feb 26, 2021 | - Healthcare workers and staff involved in anti-epidemic work - Persons aged 60 or above and a maximum of 2 carers accompanying older adults aged above 70 - Residents and staff of residential care homes for the elderly and persons with disabilities - People providing essential public services - People providing cross-boundary transportation or working at control points and ports |
| Second^2^ | Mar 8, 2021 | - Staff of food and beverages premises, markets, supermarkets, convenience stores, couriers, and takeaway delivery - Staff of local public transport operators - Registered construction workers - Property management staff - Teachers and school staff - Staff in the tourism industry - Staff of scheduled premises under the Prevention and Control of Disease |
| Third^3^ | Mar 16, 2021 | - People aged between 30 and 59 - Students aged 16 or above studying outside Hong Kong - Domestic helpers |
| Fourth^4^ | Apr 15, 2021 | - People aged ≥16 can receive BNT162b2 - People aged ≥18 can receive CoronaVac |
| Fifth^5^ | Jun 10, 2021 | - People aged ≥12 can receive BNT162b2 |
| Sixth^6^ | Nov 11, 2021 | - Eligible persons under certain groups can receive a third dose of COVID-19 vaccine free of charge |
| Seventh^7^ | Nov 20, 2021 | - People aged 12 to 17 can receive CoronaVac |
| Eighth^8^ | Nov 23, 2021 | - Members of the public who have received two doses of the CoronaVac vaccine with the second dose received 6 months ago can make reservations for a third dose of a COVID-19 vaccine irrespective of certain groups |
| Ninth^9^ | Jan 21, 2022 | - People aged 5 to 11 can receive CoronaVac |
| Tenth^9^ | Feb 16, 2022 | - People aged 5 to 11 can receive BNT162b2 |
| Eleventh^10^ | Feb 15, 2022 | - People aged 3 to 4 can receive CoronaVac |
| Twelfth^11^ | Mar 05, 2022 | - People aged ≥60 can receive a third dose at 3 months (reduced from 6 months) after the second dose - People aged 5 to 17 can receive a second dose of BNT162b2 at 8 weeks (reduced from 12 weeks) after the first dose - Immunocompromised people aged ≤12 can make reservations for a third dose of a COVID-19 vaccine 4 weeks after receiving the first two doses |
| Thirteenth^9,10^ | Mar 11, 2022 | - People who received two doses of CoronaVac can receive a third dose of CoronaVac or BioNTech vaccine 3 months after the second dose - People who received two doses of BioNTech can receive a third dose of BioNTech or CoronaVac vaccine 5 months after the second dose - People aged 12 to 17 who have received two doses of BioNTech vaccine can receive a third dose of BioNTech or CoronaVac vaccine 5 months after the second dose |
| Fourteenth^9^ | Mar 21, 2022 | - Immunocompromised people aged ≥12 who have received three doses of COVID-19 vaccine may receive a fourth vaccine dose at least 3 months after their last dose |
| Fifthteenth^11^ | Apr 14, 2022 | - Children aged 3 to 11 who have received two doses of CoronaVac can receive a third dose after 3 months - Persons aged 60 or above who have received three doses of the CoronaVac or BioNTech vaccine can receive a fourth dose at least 3 months after the last dose |
| Sixteenth^12^ | May 21, 2022 | - Uninfected individuals aged 18 to 59 who are at higher risk of COVID-19 exposure or have personal needs may choose to receive a fourth dose of COVID-19 vaccine, regardless of whether they have received BioNTech or CoronaVac vaccine as their previous doses |
| Seventeenth^13^ | Aug 4, 2022 | - Children aged 6 months to 3 years can receive the CoronaVac vaccine - Persons aged 50 to 59 who have received three doses of CoronaVac or BioNTech vaccine can receive a fourth dose at least 3 months after the last dose |

**References:**

1. HKSAR. Government announces 2019 COVID-19 Vaccination Programme. Press Releases. 18 Feb 2021 (https://www.info.gov.hk/gia/general/202102/18/P2021021800767.htm?fontSize=1)

2. HKSAR. Government expands scope of priority groups and opens more CVCs. Press Releases. 8 Mar 2021 (https://www.info.gov.hk/gia/general/202103/08/P2021030800738.htm)

3. HKSAR. Vaccination priority groups to be expanded to cover people aged 30 or above. Press Releases. 15 Mar 2021 (https://www.info.gov.hk/gia/general/202103/15/P2021031500626.htm?fontSize=1)

4. HKSAR. COVID-19 Vaccination Programme opens to persons aged 16 or above. Press Releases. 15 Apr 2021 (https://www.info.gov.hk/gia/general/202104/15/P2021041500565.htm?fontSize=1)

5. HKSAR. Persons aged 12 to 15 can make reservations to receive BioNTech vaccine from tomorrow. Press Releases. 10 Jun 2021 (https://www.info.gov.hk/gia/general/202106/10/P2021061000556.htm?fontSize=1)

6. Third dose COVID-19 vaccination arrangements for persons under certain groups. Press Releases. 3 Nov 2021 (https://www.info.gov.hk/gia/general/202111/03/P2021110300536.htm)

7. SFH approves lowering age limit for receiving CoronaVac vaccine. Press Releases. 20 Nov 2021 (https://www.info.gov.hk/gia/general/202111/20/P2021112000292.htm)

8. Government extends third dose COVID-19 vaccination arrangements. Press Releases. 18 Nov 2021 (https://www.info.gov.hk/gia/general/202111/18/P2021111800310.htm)

9. HKSAR. Arrangements for children aged 5 to 11 to receive COVID-19 vaccines. Press Releases. 20 Jan 2022 (https://www.info.gov.hk/gia/general/202201/20/P2022012000714.htm)

10. HKSAR. Lowering of minimum age for receiving Sinovac vaccine to three years old starting from February 15. Press Releases. 13 Feb 2022 (<https://www.info.gov.hk/gia/general/202202/13/P2022021300644.htm>)

11. HKSAR. Third dose Sinovac vaccine booking arrangements for children aged 3 to 11. Press Releases. 13 Apr 2022 (<https://www.info.gov.hk/gia/general/202204/13/P2022041300609.htm>)

12. HKSAR. Persons aged 18 to 59 may choose to receive fourth dose of COVID-19 vaccine. Press Releases. 21 May 2022 (<https://www.info.gov.hk/gia/general/202205/21/P2022052000831.htm>)

13. HKSAR. COVID-19 vaccination arrangements for children aged six months or above and for persons aged from 50 to 59 receiving fourth dose Press Releases. 2 Aug 2022 (https://www.info.gov.hk/gia/general/202208/02/P2022080200699.htm)

**Supplementary Table 2.** Conditions used to define severe COVID-19

| **Diagnosis / Procedures** | **ICD-9-CM diagnosis / procedure code** |
| --- | --- |
| Viral Encephalitis / Acute Necrotizing Encephalitis | 049.8 |
| Encephalitis in viral disease | 323.0 |
| Late effect of viral encephalitis | 139.0 |
| Meningoencephalitis | 322.9, 323.0, 323.4-323.9, 330.8, 377.73, 046.3, 049.0, 049.8, 049.9, 036.1, 056.01, 136.2, 130.x, 054.3, 094.1, 072.2, 013.0, 062.4, 045.0, 062.x |
| Pneumonia (COVID-19) | 519.8 |
| Croup | 464.4 |
| Seizure | 333.2, 345.x, 649.4, 780.3, 779.0, 780.3 |
| Febrile convulsion | 780.31 |
| Status epilepticus | 345.3 |
| Ventilatory support | 39.65, 89.18, 93.90, 93.95, 93.96, 96.7, 96.04 |
| High-flow oxygen | 93.96 |
| Non-invasive mechanical ventilation | 93.99 |
| Invasive mechanical ventilation | 96.70 |
| BiPAP | 93.90 |
| Multisystem inflammatory syndrome in children (MIS-C) | 446.1 |
| MRI brain | 88.91 |
| CT brain | 87.03 |
| Lumbar puncture | 03.31 |
| EEG | 89.14 |

BiPAP: bilevel positive airway pressure; CT: computer tomography; EEG: electroencephalogram; MRI: magnetic resonance imaging

**Supplementary Table 3**. Summary of COVID-19-related death and ICU admission/ventilatory support cases

| **Outcomes** | **COVID-19-related death** | **COVID-19-related ICU admission or ventilatory support** |
| --- | --- | --- |
| Number of cases | 8 | 99 |
| Age, years (mean (SD)) | 8.75 (5.06) | 8.24 (4.23) |
| Sex, male (%) | 4 (50.0) | 60 (60.6) |
| **Vaccination status - no. (%)** |  |  |
| Unvaccinated | 6 (75.0) | 57 (57.6) |
| 1 dose BNT162b2 | 1 (12.5) | 7 (7.1) |
| 1 dose CoronaVac | 1 (12.5) | 16 (16.2) |
| 2 doses BNT162b2 | 0 (0.0) | 8 (8.1) |
| 2 doses CoronaVac | 0 (0.0) | 10 (10.1) |
| 3 doses BNT162b2 | 0 (0.0) | 1 (1.0) |
| 3 doses CoronaVac | 0 (0.0) | 0 (0.0) |

ICU: intensive care unit; SD: standard deviation

**Supplementary Table 4**. Sensitivity analysis: exclusion of cases and controls who received their last vaccine dose for more than 90 days

| **Vaccination status** | **Case** | **Control** | **Crude OR (95% CI)** | **Adjusted OR (95% CI)** | **VE % (95% CI)** |
| --- | --- | --- | --- | --- | --- |
| *COVID-19* | | | | | |
| Unvaccinated | 17545 | 45461 | (Ref) | (Ref) | (Ref) |
| *1 dose* |  |  |  |  |  |
| BNT162b2 | 1779 | 5900 | 0.559 (0.525 – 0.595) | 0.552 (0.518 – 0.587) | 44.8 (41.3; 48.2) |
| CoronaVac | 5855 | 18577 | 0.795 (0.766 – 0.824) | 0.788 (0.760 – 0.817) | 21.2 (18.3; 24.0) |
| *2 doses* |  |  |  |  |  |
| All BNT162b2 | 730 | 1758 | 0.481 (0.433 – 0.534) | 0.470 (0.423 – 0.522) | 53.0 (47.8; 57.7) |
| All CoronaVac | 1279 | 3534 | 0.635 (0.588 – 0.685) | 0.626 (0.580 – 0.675) | 37.4 (32.5; 42.0) |
| *3 doses* |  |  |  |  |  |
| All BNT162b2 | 190 | 515 | 0.342 (0.277 – 0.423) | 0.337 (0.273 – 0.416) | 66.3 (58.4; 72.7) |
| All CoronaVac | 103 | 165 | 0.690 (0.524 – 0.908) | 0.681 (0.518 – 0.896) | 31.9 (10.4; 48.2) |
| B-B-C | 36 | 56 | 0.589 (0.376 – 0.923) | 0.582 (0.371 – 0.912) | 41.8 (8.8; 62.9) |
| C-C-B | 1 | 4 | 0.261 (0.028 – 2.390) | 0.257 (0.028 – 2.356) | 74.3 (-135.6; 97.2) |
| *COVID-19-related hospitalization* | | | | | |
| Unvaccinated | 1188 | 10222 | (Ref) | (Ref) | (Ref) |
| *1 dose* |  |  |  |  |  |
| BNT162b2 | 70 | 671 | 0.620 (0.470 – 0.818) | 0.642 (0.486 – 0.849) | 35.8 (15.1; 51.4) |
| CoronaVac | 275 | 2483 | 0.952 (0.820 – 1.106) | 0.962 (0.827 – 1.119) | 3.8 (-11.9; 17.3) |
| *2 doses* |  |  |  |  |  |
| All BNT162b2 | 39 | 327 | 0.436 (0.292 – 0.649) | 0.467 (0.312 – 0.699) | 53.3 (30.1; 68.8) |
| All CoronaVac | 135 | 1136 | 0.761 (0.611 – 0.948) | 0.785 (0.629 – 0.979) | 21.5 (2.1; 37.1) |
| *3 doses* |  |  |  |  |  |
| All BNT162b2 | 18 | 164 | 0.278 (0.151 – 0.512) | 0.296 (0.160 – 0.548) | 70.4 (45.2; 84.0) |
| All CoronaVac | 12 | 73 | 0.492 (0.252 – 0.964) | 0.491 (0.248 – 0.973) | 50.9 (2.7; 75.2) |
| B-B-C | 8 | 14 | 1.649 (0.613 – 4.432) | 1.790 (0.671 – 4.771) | -79.0 (-377.1; 32.9) |
| C-C-B | 0 | 1 | - | - | - |
| *Severe COVID-19* | | | | | |
| Unvaccinated | 1043 | 9108 | (Ref) | (Ref) | (Ref) |
| *1 dose* |  |  |  |  |  |
| BNT162b2 | 55 | 523 | 0.640 (0.468 – 0.874) | 0.647 (0.472 – 0.887) | 35.3 (11.3; 52.8) |
| CoronaVac | 233 | 2153 | 0.936 (0.795 – 1.103) | 0.951 (0.806 – 1.121) | 4.9 (-12.1; 19.4) |
| *2 doses* |  |  |  |  |  |
| All BNT162b2 | 30 | 283 | 0.358 (0.228 – 0.562) | 0.377 (0.240 – 0.593) | 62.3 (40.7; 76.0) |
| All CoronaVac | 111 | 876 | 0.844 (0.659 – 1.080) | 0.856 (0.668 – 1.097) | 14.4 (-9.7; 33.2) |
| *3 doses* |  |  |  |  |  |
| All BNT162b2 | 13 | 106 | 0.282 (0.138 – 0.576) | 0.299 (0.146 – 0.614) | 70.1 (38.6; 85.4) |
| All CoronaVac | 12 | 76 | 0.517 (0.268 – 0.998) | 0.506 (0.260 – 0.985) | 49.4 (1.5; 74.0) |
| B-B-C | 6 | 14 | 0.946 (0.328 – 2.731) | 0.995 (0.341 – 2.899) | 0.5 (-189.9; 65.9) |
| C-C-B | 0 | 1 | - | - | - |

OR: odds ratio, VE: vaccine effectiveness, CI: confidence interval, B-B-C: two doses of BNT162b2 followed by CoronaVac, C-C-B: two doses of CoronaVac followed by BNT162b2

**Supplementary Table 5**. Sensitivity analysis: inclusion of RAT-positive cases

| **Vaccination status** | **Case** | **Control** | **Crude OR (95% CI)** | **Adjusted OR (95% CI)** | **VE % (95% CI)** |
| --- | --- | --- | --- | --- | --- |
| *COVID-19* | | | | | |
| Unvaccinated | 28821 | 26394 | (Ref) | (Ref) | (Ref) |
| *1 dose* |  |  |  |  |  |
| BNT162b2 | 8004 | 8644 | 0.737 (0.707 – 0.768) | 0.726 (0.696 – 0.756) | 27.4 (24.4; 30.4) |
| CoronaVac | 12672 | 13388 | 0.851 (0.826 – 0.878) | 0.843 (0.818 – 0.870) | 15.7 (13.0; 18.2) |
| *2 doses* |  |  |  |  |  |
| All BNT162b2 | 10297 | 11103 | 0.684 (0.654 – 0.716) | 0.672 (0.643 – 0.703) | 32.8 (29.7; 35.7) |
| All CoronaVac | 7243 | 7021 | 0.843 (0.804 – 0.884) | 0.837 (0.798 – 0.877) | 16.3 (12.3; 20.2) |
| *3 doses* |  |  |  |  |  |
| All BNT162b2 | 1419 | 1848 | 0.494 (0.450 – 0.542) | 0.489 (0.445 – 0.536) | 51.1 (46.4; 55.5) |
| All CoronaVac | 533 | 574 | 0.710 (0.625 – 0.806) | 0.701 (0.617 – 0.796) | 29.9 (20.4; 38.3) |
| B-B-C | 169 | 170 | 0.645 (0.515 – 0.806) | 0.634 (0.507 – 0.794) | 36.6 (20.6; 49.3) |
| C-C-B | 3 | 19 | 0.123 (0.036 – 0.417) | 0.122 (0.036 – 0.413) | 87.8 (58.7; 96.4) |
| *COVID-19-related hospitalization* | | | | | |
| Unvaccinated | 1211 | 11357 | (Ref) | (Ref) | (Ref) |
| *1 dose* |  |  |  |  |  |
| BNT162b2 | 163 | 1719 | 0.734 (0.602 – 0.894) | 0.770 (0.631 – 0.940) | 23.0 (6.0; 36.9) |
| CoronaVac | 307 | 3023 | 0.940 (0.817 – 1.082) | 0.960 (0.834 – 1.106) | 4.0 (-10.6; 16.6) |
| *2 doses* |  |  |  |  |  |
| All BNT162b2 | 212 | 2585 | 0.576 (0.473 – 0.703) | 0.611 (0.501 – 0.747) | 38.9 (25.3; 49.9) |
| All CoronaVac | 356 | 3008 | 1.058 (0.903 – 1.241) | 1.081 (0.921 – 1.269) | -8.1 (-26.9; 7.9) |
| *3 doses* |  |  |  |  |  |
| All BNT162b2 | 31 | 534 | 0.351 (0.230 – 0.537) | 0.373 (0.244 – 0.569) | 62.7 (43.1; 75.6) |
| All CoronaVac | 15 | 224 | 0.550 (0.319 – 0.949) | 0.558 (0.323 – 0.965) | 44.2 (3.5; 67.7) |
| B-B-C | 10 | 49 | 1.276 (0.626 – 2.601) | 1.322 (0.647 – 2.702) | -32.2 (-170.2; 35.3) |
| C-C-B | 0 | 6 | - | - | - |
| *Severe COVID-19* | | | | | |
| Unvaccinated | 1049 | 10030 | (Ref) | (Ref) | (Ref) |
| *1 dose* |  |  |  |  |  |
| BNT162b2 | 129 | 1349 | 0.765 (0.612 – 0.957) | 0.791 (0.632 – 0.989) | 20.9 (1.1; 36.8) |
| CoronaVac | 252 | 2639 | 0.905 (0.774 – 1.057) | 0.920 (0.787 – 1.075) | 8.0 (-7.5; 21.3) |
| *2 doses* |  |  |  |  |  |
| All BNT162b2 | 166 | 2089 | 0.592 (0.474 – 0.739) | 0.617 (0.493 – 0.771) | 38.3 (22.9; 50.7) |
| All CoronaVac | 296 | 2301 | 1.253 (1.048 – 1.497) | 1.280 (1.070 – 1.531) | -28.0 (-53.1; -7.0) |
| *3 doses* |  |  |  |  |  |
| All BNT162b2 | 22 | 336 | 0.434 (0.263 – 0.716) | 0.454 (0.275 – 0.751) | 54.6 (24.9; 72.5) |
| All CoronaVac | 12 | 189 | 0.560 (0.304 – 1.029) | 0.567 (0.308 – 1.044) | 43.3 (-4.4; 69.2) |
| B-B-C | 6 | 29 | 1.366 (0.546 – 3.413) | 1.387 (0.553 – 3.481) | -38.7 (-248.1; 44.7) |
| C-C-B | 0 | 1 | - | - | - |

OR: odds ratio, VE: vaccine effectiveness, CI: confidence interval, B-B-C: two doses of BNT162b2 followed by CoronaVac, C-C-B: two doses of CoronaVac followed by BNT162b2
